# ‘Hairy’ root extracts as source for ‘green’ synthesis of silver nanoparticles and medical applications†

**DOI:** 10.1039/d0ra07784d

**Published:** 2020-10-27

**Authors:** Natalia Kobylinska, Anatolij Shakhovsky, Olena Khainakova, Dmytro Klymchuk, Liliya Avdeeva, Yakiv Ratushnyak, Volodymyr Duplij, Nadiia Matvieieva

**Affiliations:** Taras Shevchenko National University of Kyiv 64 Volodymyrska Str. Kyiv 01601 Ukraine kobilinskaya@univ.kiev.ua; Institute of Cell Biology and Genetic Engineering, NAS of Ukraine 148 Zabolotnogo Str. Kyiv 03143 Ukraine; University of Oviedo 8 Julián Claveria Av. Oviedo 33006 Spain; M.G. Kholodny Institute of Botany, NAS of Ukraine 2 Tereshchenkivska Str. Kyiv 02000 Ukraine; Zabolotny Institute of Microbiology and Virology, NAS of Ukraine 154 Zabolotnogo Str. Kyiv 03143 Ukraine

## Abstract

The research was focused on the synthesis of silver nanoparticles (AgNPs) using extracts from the “hairy” root cultures of *Artemisia tilesii* Ledeb. and *Artemisia annua* L. The effect of operational parameters such as type of solvent, temperature of extraction, flavonoids concentration, and reducing power of the wormwood “hairy” root extracts on the particle size and yield of the resultant nanoparticles is reported for the first time. From the studied solvents, a water–ethanol mixture with a concentration of 70 vol% was found to be the best for the extraction of flavonoids from all “hairy” root cultures. The total flavonoid contents in *A. annua* and *A. tilesii* “hairy” root extracts were up to 80.0 ± 0.9 and 108 ± 4.4 mg RuE per g DW, respectively. Identification of flavonoids was confirmed by UPLC-ESI-UHR-Qq-TOF-MS analysis. Luteolin-7-β-d-glucopyranosid, isorhamnetin 3-*O*-glucoside, baicalein-7-*O*-glucuronide, apigenin-7-*O*-glucoside, quercetin, sitosterol, caffeoylquinic, galic, chlorogenic and caffeic acids were founded in the extracts. These extracts demonstrated the high reducing activities. Spherical, oval and triangular nanoparticles with effective sizes of 5–100 nm were observed. The TEM data revealed great differences in the shapes of NPs, obtained from the extracts from different root clones. The clustered and irregular NPs were found in the case of using ethanol extracts, mostly aggregated and having the size of 10–50 nm. The sizes of AgNPs decreased to 10–30 nm in the case of using aqueous extracts obtained at 80 °C. Biosynthesized AgNPs showed surface plasmon resonance in the range of 400–450 nm. The antimicrobial activity of the as-produced AgNPs was studied by disc diffusion method on Gram-positive (*Staphylococcus aureus* ATCC 25923 (F-49)), Gram-negative (*Pseudomonas aeruginosa* ATCC 27853 (F-51), *Escherichia coli* ATCC 25922 (F-50)) and *Candida albicans* ATCC 88-653 strains. It was found that the nanoparticles in some cases possessed the greater ability to inhibit microorganism growth compared to 1 mM AgNO_3_ solution. The colloidal solutions of the obtained AgNPs were stable in the dark for 12 months at room temperature. Thus, the *A. annua* and *A. tilesii* “hairy” root extracts can be used for obtaining of bioactive AgNPs.

## Abstract 2

The research was focused on the synthesis of silver nanoparticles (AgNPs) using extracts from the “hairy” root cultures of *Artemisia tilesii* Ledeb. and *Artemisia annua* L.

## Graphical abstract

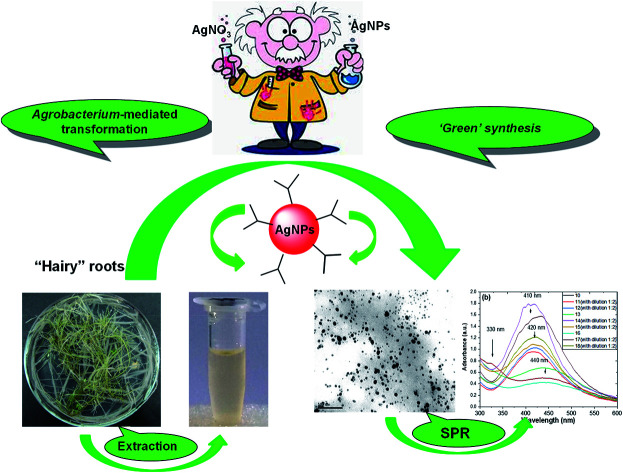

## 1 Introduction

Nowadays, there are avalanche of investigations carried worldwide, aimed at the synthesis of nanosized inorganic (metal, magnetic, oxides, *etc.*) and organic materials because of the striking difference in the physical, chemical, and optical characteristics compared to the bulk materials.^1^ At the same time, there is considerable interest in obtaining noble metal nanoparticles (NPs), especially from silver and gold. The small size makes the penetration of these particles through a cell membrane easier. The nanosizes of NPs facilitate their homogeneous distribution in cells in the process of implication. Based on their furnished superior characteristics, there are potential applications in medicine (antimicrobial, antibacterial and anticancer properties) and drug delivery systems,^2^ as cryogenic superconducting materials,^3^ in catalysis^4^ and agriculture practices,^5^ and as biological sensors.^6^ Silver nanoparticles (AgNPs) have the most prominent antimicrobial activities, and their potential can be exploited in different fields of human activity.^7^ In this key role, the uses of AgNPs are highly dependent on their size, shape, surface structure and colloid solution stability.

Different synthesis methods can be performed to obtain AgNPs, including chemical,^8,9^ electrochemical, photochemical,^10^ and biological (using bacteria, fungi, viruses). Natural organic precursors such as plant extracts, sugars, and biodegradable polymers can also be used for the so-called “*green*” synthesis of nanoparticles.^11,12^ The last one has some advantages as opposed to the chemical approach because of the exception of using hazardous components and high energy requirements.^13^ Biosynthesized AgNPs have been used in a wide range of antibacterial and antimicrobial applications such as wound treatment,^14^ human pathogenic microorganisms^15^ and food pathogen inhibition,^11^ which is why these NPs can be used for medical purpose.^16^

Currently, many studies have been published regarding the use of plant extracts for the synthesis of AgNPs. For example, Ahmed *et al.* in 2016 (ref. 17) reviewed more than 40 plant species used for this purpose, including numerous medicinal plants: *Cymbopogon citratus*, *Ziziphora tenuior*, *Ficus carica*, *Centella asiatica*, *Coccinia indica*, *Trachyspermum ammi*, *Moringa oleifera*, *Aloe vera*, *Citrus sinensis*, *Datura metel*, and *Carica papaya*. Other authors^18^ cited more than 90 articles, where the use of plants in obtaining AgNPs was described. A comparison was performed studying the effectiveness of AgNPs synthesis after the contact of AgNO_3_ solution and different plant extracts. The effect of plant secondary metabolite content, accumulation of other biologically active compounds, Rh potential, antioxidant and reducing activity of plant extracts on AgNPs synthesis was studied. One of the disadvantages of this method is that a biological system is always less controllable compared to a chemical synthesis. In general, plant extracts usually have alkaloids, flavonoids, proteins, polysaccharides, cellulose, polyphenolic compounds and its metabolites, which could be used for the initiation of NPs synthesis.^11,19^ Used as non-toxic stabilizing or reducing agents, different compounds of plant extracts can be adsorbed onto the surface of the particles through various mechanisms.^20^ Polyphenolic compounds of the extract can coat AgNPs, and this process allows obtaining more stable particles in comparison with the one synthesized by chemical reductants such as citrate or sodium borohydride.^21^ The mechanism of the effects of the different extracted components for the reduction and stabilization of AgNPs has not yet been explained. The composition and concentration of reducing agents in plant extracts have a great influence towards the size, the shape (*e.g.*, ellipsoidal, spherical, flowers, rods, stars or triangles), the morphology and the stability of the NPs obtained during synthesis.^22^

It is known that *Agrobacterium rhizogenes rol* genes are inducers of plant secondary metabolism^23,24^ in the case of the genetic transformations and their incorporation in plant genome.^25,26^ That is why the transformed “hairy” roots carrying *A. rhizogenes rol* genes can significantly differ from the mother plants in the chemical composition and bioactivity of cell components.^27–29^ Thus, transgenic “hairy” roots, obtained after the transformation, can demonstrate greater bioactivity than the mother plants. In particular, they can significantly exceed the original non-transformed plants in their reducing activity. Numerous *Artemisia* genus plants are known for the synthesis of different bioactive compounds.^30^ However, the Aleutian mugwort plants *Artemisia tilesii* Ledeb. remain practically unknown. These plants are grown in the northern regions of America and Europe, and are used in regional traditional medicine in Alaska.^31^ In our previous study of *Artemisia* “hairy” root clones, great differences in the secondary metabolite content and corresponding bioactivity were found.^32^ These clones differed not only morphologically, but also by the level of accumulation of flavonoids.^33^ “Hairy” roots were used earlier for nanoparticle synthesis. The positive effect was demonstrated and compared, for example, with that of yeasts used for this type of process.^34^ Such results give a reason to assume the possibility of the effective use of selected “hairy” root lines, characterized by a high level of reducing activity, for the “*green*” synthesis of AgNPs. In addition, “hairy” root extracts are characterized by a high antioxidant activity due to the increased content of bioactive compounds, and could be employed in pharmaceutical, medical and cosmetic applications.

In this study, the “hairy” root cultures of *A. tilesii* and *A. annua* were used for the effective extraction of bioactive compounds and the “*green*” synthesis of AgNPs. The strategy was presented for the detection and structural elucidation of flavonoids and co-extracted compounds from a complex matrix in a single chromatographic run using ultra-performance liquid chromatography coupled with electro spray ionization time-of-flight mass spectrometry (UPLC-ESI-UHR-Qq-TOF-MS). The effect of some parameters (use of “hairy” root lines characterized by different bioactivity, the type of the solvent, and the temperature of extraction) on the particle size and on the yield of the resultant nanoparticles was studied. The produced AgNPs were tested for their antimicrobial activities. *Candida albicans*, *Staphylococcus aureus*, *Pseudomonas aeruginosa* and *Escherichia coli* were involved in this study. Therefore, to our knowledge, this is the first scientific report on the use of “hairy” root culture extracts to synthesize AgNPs in an eco-friendly way with the consequent monitoring of their stability and biocide activity against pathogenic microorganisms.

## 2 Experimental part

### 2.1 Chemicals and reagents

All chemicals used in this study were of high-quality analytical grade, and used without further purification. Silver nitrate (AgNO_3_, 99.9% Merck), sodium carbonate (Na_2_CO_3_), and aluminum chloride (AlCl_3_) (Sigma-Aldrich, USA) were used for the initiation of NPs and the total flavonoid content study. Nylon membranes were used for UPLC-ESI-UHR-Qq-TOF-MS analysis. The HPLC grade solvents used in the chromatographic analysis were obtained from Sigma-Aldrich (Spain). All aqueous solutions were prepared using double-distilled water (Milli-Q plus Millipore, France).

### 2.2 Preparation of plant extracts

“Hairy” root cultures of *A. annua* and *A. tilesii* medicinal plants (Fig. S1†) from the collection of the Laboratory of Adaptational Biotechnology of the Institute of Cell Biology and Genetic Engineering NAS of Ukraine were grown under sterile conditions on Petri dishes using a Murashige and Skoog solid nutrient medium (Duchefa, Netherlands) with twice reduced concentration (½MS). The lyophilized roots were used as the material for obtaining extracts.

#### 2.2.1 Ethanol extraction

50 mg of dried and powdered “hairy” roots of *A. tilesii* (clones no. 17, 21 and 22) and *A. annua* (clones no. 15, 17 and 19) by Retsch MM400 (Germany) were added to 5 ml of 70 vol% EtOH, and agitated during 3 days on the rotary shaker Clim-O-Shake system Kuhner IRC-1-U at 28 °C. Then, the extracts were centrifuged (Eppendorf Centrifuge 5415C, Germany), and the supernatants were used for the synthesis of nanoparticles.

#### 2.2.2 Aqueous extraction

For obtaining the aqueous extracts, two temperature modes were used. 50 mg of dried root powder was added to 5 ml of double-distilled water to obtain the first type of aqueous extract. The mixture was stirred at 28 °C on a rotary shaker Clim-O-Shake system Kuhner IRC-1-U and agitated for 3 days. For obtaining the second type of aqueous extracts, the same procedure was done with some modification. During the 3 days of incubation on the shaker, the solutions were heated on a water bath at 80 °C for 1 hour. Then, the extracts were centrifuged.

### 2.3 Preparation of AgNPs

The aqueous solution of 1 mM silver nitrate (AgNO_3_) was prepared in a 100 ml flask. 0.2 ml of the ethanol or water root extract was mixed with 2 ml of AgNO_3_ solution under magnetic stirring at 90 °C for one hour to reduce Ag^+^ to Ag^0^, and was then left at 28 °C until the colour of the mixture changed from yellowish to brown.

### 2.4 Characterisation of AgNPs

Transmission electron microscopy (TEM) was used to examine the size and morphology of the synthesized NPs. The image was done on a TEM 1230 JEOL (Tokyo, Japan) with an acceleration voltage of 80 kV. The samples for TEM investigations were prepared by drying 0.03–0.05 μl of colloidal solution dropwise on Cu-grids with a previously carbon-coated film at room temperature.

Fourier transform infrared (FTIR) spectra were recorded from the liquid samples by placing between KBr glasses using a Nicolet Nexus 470 (Thermo, USA) spectrometer.

The XRD phase identification of the obtained AgNPs was performed a PANalytical X'Pert Pro diffractometer with X-rays of wavelength (*λ*) = 1.54056 Å and operated at 40 kV. Tescan Mira 3 LMU equipment was used for Scanning Electron Microscope (SEM) measurements. The energy dispersive X-ray analysis (EDX) was performed in the same microscope equipped with an Advanced Aztec Energy (IE350)/X-max80.

UV-visible method was employed for the complex analysis of the biosynthesized colloid solutions of AgNPs. The spectra of the colloidal solution (surface plasmon resonance, SPR) were measured periodically (1, 2 and 10 days) in the wavelength range of 300–700 nm by a Fluorat-02 Panorama spectrophotometer (Russia). In some samples, the content of the obtained solution was diluted twice with double-distilled water for measurements.

### 2.5 Total flavonoid content

The total flavonoid content was measured using aluminium chloride solution by modified spectrophotometric method.^35^ Briefly, 0.25 ml sample of each extract was mixed with 1 ml of double-distilled water and 0.075 ml of 5% NaNO_2_ solution, and allowed to react for 5 min at room temperature. After that, 0.075 ml of 10% AlCl_3_ solution was added. After another 5 min of incubation, 0.5 ml of 1 M NaOH solution and 0.6 ml of double-distilled water were added to the reaction mixture, and the absorbance of the sample was measured at 510 nm. The total flavonoid content was expressed as milligrams per gram of dry root weight in rutin equivalent (mg RE per g DW). The concentration of the flavonoid compounds was calculated by using the equation that was obtained in the range from 50 to 500 μg ml^−1^ with a calibration curve: *C* (rutin) = 0.0900*x* − 0.0309 (*R*^2^ = 0.9996).

### 2.6 UPLC-ESI-UHR-Qq-TOF-MS analysis

For the analysis of multiple compositions in the ethanol extract of roots, the UPLC system (Dionex Ultimate 3000) was coupled with an Ultra-High Resolution Qq-Time-of-Flight (UHR-QqTOF) mass spectrometry equipped with an electrospray ionization (ESI) interface (Bruker Impact II). The presence of phenolic compounds in the extracts was determined based on their mass fragmentation pattern, low mass error within the acceptance range of ±5 mDa and ion response. The mass spectrometer was operated in the negative ESI mode with Duo-Spray source, and the mass scan range was set at *m*/*z* 50–2500 for the TOF-MS scan using a resolution of 2700. The following parameter conditions were used: ion spray voltage, 3500 V; ion source heater, 500 °C; curtain gas, 25 psi; collision energy, 10 eV; declustering potential 100. The analyst TF software (version 1.7) combined with the information-dependent acquisition packing was used to acquire the MS data. The mobile phase was composed of 0.1% formic acid in water (elution A) and methanol (elution B) using a gradient elution of 30% elution B (0–5 minutes), from 30% to 50% of elution B (5 and 20 minutes), from 50% to 90% elution B (20–40 minutes), and from 90% to 100% of elution B (40–45 minutes).

### 2.7 Reducing power assay

This method was adapted from a previous study^36^ with slight modifications to determine the ability of extracts to reduce Fe^3+^ ions. For this study, 0.016–0.062 ml of the extract was mixed with 0.312 ml of 0.2 M phosphate buffer (pH 6.6) and 0.312 ml of 1% potassium ferricyanide, and incubated at 50 °C. 0.312 ml of 10% trichloroacetic acid solution was added after 30 min incubation. After this procedure, 1.25 ml of the supernatant was mixed with 1.25 ml of distilled water and 0.25 ml of 0.1% ferric chloride. The absorbance was measured at 700 nm. Equivalent concentrations (EC_0.5_) were calculated as the amount of dried root material needed to obtain absorbance = 0.5 on the basis of the equations expressing the optical density dependence on the concentration of the extract. The equations were obtained by the linear regression method. Rutin at the concentration of 1 mg l^−1^ was used as a positive control.

### 2.8 Statistical analysis

The analytical data were shown as the means of triplicates, and subjected to variance analysis (ANOVA). The results were expressed as the mean ± standard deviation. The data analysis was conducted using Tukey's method in multiple comparisons. It is considered to have significant difference when a level of *p* < 0.05 was achieved. The linear regression method was applied and the coefficient of determination (*R*^2^) was calculated for establishing the relationship between the values.

### 2.9 Antimicrobial activity assay

The antimicrobial activity of the synthesized AgNPs was studied by the disc diffusion method.^37^*Staphylococcus aureus* ATCC 25923, *Escherichia coli* ATCC 25922, *Pseudomonas aeruginosa* ATCC 27853 and *Candida albicans* ATCC 88-653 strains were used as the test microorganisms. The Gram-positive and Gram-negative bacteria were pre-cultured in Mueller–Hinton broth overnight at 37 °C. Afterward, each strain was adjusted at a concentration of 10^8^ cells ml^−1^ using 0.5 McFarland standard. The *C. albicans* inoculum was prepared from the 48 h culture of isolates in potato dextrose broth. The 20 μl of the AgNPs solutions was applied on standard 6 mm diameter filter paper disks. The disks without nanoparticles, but with ciprofloxacin, were treated as a positive control for the evaluation of microorganism growth inhibition. The filter paper disks with 1 mM solution of AgNO_3_ were used as a negative control. The disks impregnated with AgNPs, AgNO_3_ and ciprofloxacin were placed on Mueller–Hinton agar in Petri dishes, and incubated for 24 h at 35 °C prior to determination of the results. The antimicrobial activity was detected by measuring the zone of growth inhibition (including the disk diameter), which appeared after the incubation period.

## 3 Result and discussions

### 3.1 Preparation and characterization of “hairy” root extracts

In general, the different techniques for the extraction of bioactive compounds from medicinal plants to obtain the optimum concentrations of target components in the extract include pressurized liquid–liquid extraction, microwave- and ultrasound-assisted extraction, and supercritical fluid extraction.^38^ Usually, a dried powder of plant material is employed to extract target compounds and eliminate the interference of water at the same time. Elevated temperature, mechanical stirring or shaking, nature and quantity of solvent are the parameters that are most important for the effective extraction of the plant cell compounds. In addition, the extraction of the solute depends on the solubility of the plant origin components. Ethanol and methanol are commonly used for extraction of most flavonoids from plant tissues, as reviewed Pietta,^39^ while water is often used to extract highly polar compounds.^40^ Ethanol is often preferred, probably due to its accessibility, safety for humans, and efficiency of the extraction. Therefore, the optimum extraction conditions (EtOH and water as solvents, temperature) were used to obtain the enriched content of bioactive compounds in extracts from “hairy” roots.

#### 3.1.1 Total flavonoid contents

**Fig. 1:**
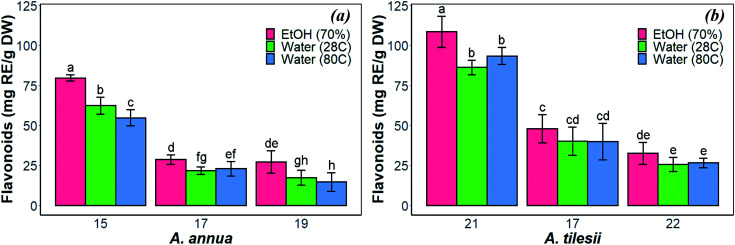
Effect of extraction condition on the total amount of flavonoids in the “hairy” roots of *Artemisia annua* (a) and *A. tilesii* (b).

Chemical analysis of ethanol and aqueous extracts of different *Artemisia* spp. showed the accumulation of several active constituents such as flavonoid, terpene, protein, polysaccharide, phenolic, coumarin and alkaloid compounds.^41^*A. annua* plants are well known as producers of artemisinin.^42,43^ However, there is a deficit of studies regarding the chemical composition of *A. tilesii* plants. In our research, the flavonoid content of the extracts of transgenic *Artemisia* roots in terms of mg rutin equivalent (RE) per g was studied. The experimental design was analyzed with the corresponding condition variables for optimizing the extraction process based on the total amount of flavonoids in the ‘hairy’ roots (Fig. 1).

As shown in Fig. 1, the content of flavonoids was very different in the extracts of *A. annua* and *A. tilesii*. The results ranged from 25.6 ± 2.08 (no. 22 roots with aqueous extraction at 28 °C) to 108.48 ± 4.45 mg RE per g DW (no. 21 roots, EtOH extraction) in *A. tilesii*. With regard to their concentrations (Fig. 1b), we detected an increased amount of flavonoids in all “hairy” root clones in the ethanol extracts compared with all kinds of aqueous extracts. In addition, the differences in the total flavonoid content in various root lines were observed. The “hairy” root no. 21 extract showed the highest flavonoid content by both EtOH and water extraction (Fig. 1b). The same data were obtained in the study of the *A. annua* (Fig. 1a) “hairy” root lines. Roots no. 15 accumulated flavonoids in greater value than the other clones. Overall, the present study, as well as our earlier reports, confirms that *A. tilesii* “hairy” root extracts were rich with flavonoids, and they could have good potential for the reduction and stabilization of metal nanoparticles during its biosynthesis.

#### 3.1.2 UPLC-ESI-UHR-Qq-TOF-MS profiles of phenolic acid and flavonoids

**Fig. 2:**
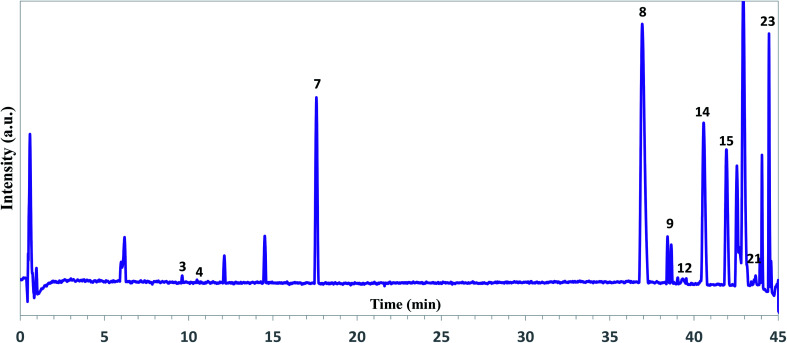
Representative total ion chromatograms of the ethanol extracts of *A. tilesii* roots obtained by UPLC-ESI-UHR-Qq-TOF-MS in the negative ion mode.

Among the available chromatography-based techniques, UPLC-ESI-UHR-Qq-TOF-MS was found to be a promising analytical technique for the chemical screening of the main components in the plant extracts.^40^ Appling this technique, it was possible to simultaneously detect and identify the bioactive compounds in the “hairy” root extracts. Negative mode ((−)ESI-MS) was run to detect and identify the bioactive compounds (Fig. 2).

As can be seen from Fig. 2, the extract profile exhibited high-resolution patterns for all peaks. A complete chromatographic separation of the main extract components was finished within 45 minutes. The presence of phenolic compounds was determined based on their mass fragmentation pattern and ion response (Fig. S2†). All motives in the MS spectra were evaluated by mass fragment with the verification process for the main components. Flavones and their derivate, phenolic acids, were the most identified compounds in the *A. tilesii* and *A. annua* extracts (Table 1). A peak at the retention time (RT) 39.3 min exhibited a [M + H]^+^ ion at *m*/*z* 170, and was identified as galic acid^44^ (Table 1). In fact, peak 14 at 40.6 min with an ion at *m*/*z* 300.1751 was assigned as quercetin. The obtained *m*/*z* values agree with the published data.^45^ Among the amino acids, the individual arginine was found in extracts. As far as the flavonoid family is concerned, we were able to characterize and identify the following compounds: luteolin-7-β-d-glucopyranosid, isorhamnetin 3-*O*-glucoside, baicalein-7-*O*-glucuronide, and apigenin-7-*O*-glucoside. Therefore, individual flavonoids and their sugar derivates (*e.g.*, glucose, glucopyranose), and sucrose were detected in the extract. In addition, two terpenes in the extracts were detected as Sterebin J and Sterebin I.

**Table 1:** Identification of bioactive compounds of the ethanol extracts of *A. tilesii* and *A. annua* “hairy” roots by UPLC-ESI-UHR-Qq-TOF-MS

Compounds	Observed molecular ion in MS (*m*/*z*)	Peaks in the MS spectra			
		*A. tilesii*		*A. annua*	
		RT, min	Present	RT, min	Present
Caffeic acid	169.8956	—	−	38.7	+
Chlorogenic acid	353.2015	41.9	+	41.7	+
Caffeoylquinic acid	353.1438	43.9	+	43.9	+
Galic acid	170.0241	39.3	+	39.3	+
Quercetin, [M − H]^−^	300.1751	40.6	+	40.6	+
Luteolin-7-β-d-glucopyranoside	475.1245	14.5	+	14.5	+
Arginine	269.1486	36.9	+	37.0	+
Isorhamnetin 3-*O*-glucoside, [M + H]^+^	476.2794	38.4	+	—	−
Baicalein-7-*O*-glucuronide	445.1860	17.6	+	—	−
Sucrose, [M + K]^+^	381.1744	44.6	+	44.5	+
Sitosterol, [M − H_2_O]^+^	397.2278	42.5	+	42.7	+
Caffeoylshiqimic acids [M + H]^+^ and Sterebin J/Sterebin I, M^+^	327.1280	43.7	+	43.7	+
Kaempferol-3-*O*-galactoside-rhamnoside-7-*O*-rhamnoside	739.1577	45.0	+	—	−
Apigenin-7-*O*-glucoside	433.1140	12.1	+	11.9	+

As one can see from Table 1, *A. tilesii* and *A. annua* extracts have a similar set of bioactive compounds, but differ from each other by the prevalence of flavonoids or phenolic acids. Briefly, the phenolic acids, caffeoylquinic acid (peak 21) and galic acid (peak 12) were present in two samples (Fig. 2). The table data also showed the presence of chlorogenic acid in both extracts. Caffeic acid exhibited a low peak only in *A. annua*. These results suggest that the distribution of phenolic acids in the transgenic roots may be influenced by the nature of the “hairy” roots or extraction conditions, but the chemical composition would always be constant within one “hairy” root line.^30^

The compounds identified in the extracts of *A. annua* and *A. tilesii* “hairy” roots are known for their medical application. For example, the anti-diabetic and anti-inflammatory activity of sitosterol was studied.^46^ The positive effects of apigenin in the treatment of diabetes, neurodegenerative disorders such as Alzheimer's and Parkinson's diseases, depression, and insomnia have been reported. This compound also demonstrated antioxidant, anti-inflammatory, antiamyloidogenic and anticancer activities.^47,48^ It should be noted that different phenolic compounds are associated with antioxidant, antidiabetic, anti-inflammatory, and anticancer activities.^49^ Terpenoids have also been reported to play an essential role in human health based on their anticancer, antioxidant, antibacterial, and antiatherosclerotic properties.^50^ Quercetin is known for its anti-inflammatory, antioxidant, anticancer, and antidiabetic effects.^39,51^ Chlorogenic acid is naturally accumulated in green coffee and tea, and is a strong antioxidant that possesses antibacterial, hepato- and cardioprotective, anti-inflammatory, neuroprotective, antiviral, and antimicrobial properties.^52^ Thus, the accumulated complex of bioactive compounds in the extracts of *A. tilesii* and *A. annua* ‘hairy’ roots is of great interest for medical application because of the potential therapeutic effects.

#### 3.1.3 Reducing power

**Fig. 3:**
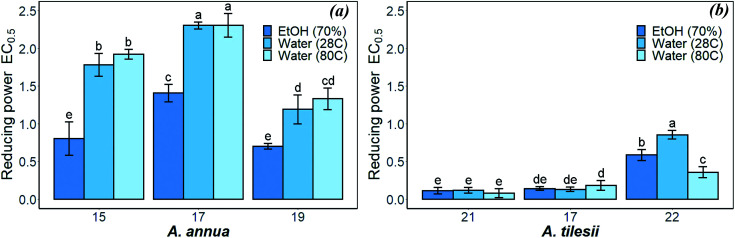
Reducing power of *A. annua* (a) and *A. tilesii* (b) “hairy” root extracts obtained under different conditions.

The reducing power is associated with antioxidant activity, and may serve as a significant reflection of this activity.^36^ The antioxidant compounds are responsible for the reduction of the ferric (Fe^3+^) form to the ferrous (Fe^2+^) form. The ability of the extract to reduce Fe suggests that they include donors of electrons, which can react with free radicals to convert them into more stable products and terminate the radical chain reaction. As shown in Fig. 3, all “hairy” root extract samples showed significant reducing activity. The values of the reducing power ranged from 0.08 to 0.8 EC_0.5_ and 0.7 to 2.3 EC_0.5_ for *A. tilesii* and *A. annua* “hairy” root extracts, respectively. The highest reducing power was observed in *A. tilesii* aqueous extracts (0.08 EC_0.5_) (Fig. 3b).

The reducing power of the *A. tilesii* extracts (Fig. 3b) is correlated with the total flavonoid content (Fig. 1b). For example, the EtOH extract of root (line no. 21) was characterized by a greater content of flavonoids. At the same time, it demonstrated the greater reducing power according to the EC_0.5_ parameter. Moreover, the “hairy” root extracts contained a complex of phenolic acids (Table 1). The phenolic compounds possess redox properties, which act as reducing agents, and were also found to be typical antioxidants. Nevertheless, the water extract (line no. 22) with the lowest flavonoids content possessed the lower reducing power. It must be noted that the reducing power of some studied *A. annua* extracts, especially water extracts, was lower than the same activity of the *A. tilesii* extracts. So, it was of great interest to compare the possibility of the *A. annua* and *A. tilesii* extracts to reduce Ag^+^, and to synthesize AgNPs because of their different reducing powers.

### 3.2 Preparation and characterization of AgNPs

**Fig. 4:**
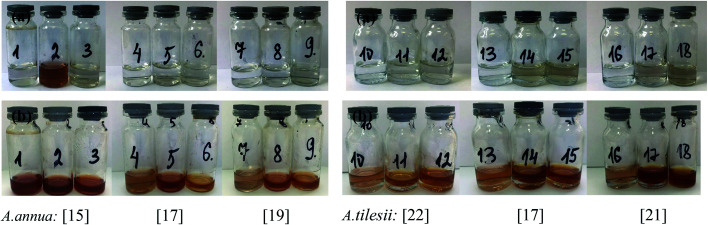
Colour changes of the mixtures of extracts with AgNO_3_ solution: in one hour (a) and after synthesis process in 24 hours (b) by *Artemisia annua* [no. 15, 17 and 19] and *A. tilesii* [no. 17, 21 and 22] “hairy” root lines with various extraction conditions: 1, 4, 7, 10, 13 and 16 – EtOH (70 vol%), 2, 5, 8, 11, 14 and 17 – H_2_O at 28 °C, and 3, 6, 9, 12, 15 and 18 – H_2_O with additional heating at 80 °C.

The effect of some physicochemical factors, such as solvent type and temperature of extraction, on the formation of AgNPs was studied. These factors also have a great influence on the size of the nanoparticles obtained in the synthesis. The use of “hairy” root extracts, which are able to reduce silver(i) ions, is a novel strategy for the synthesis of nanomaterials. In our study, the “hairy” root extracts were used to synthesize silver nanoparticles under soft condition by the addition of EtOH (70%) extracts, aqueous extracts obtained at 28 °C, aqueous extracts heated at 80 °C, and 1 mM silver nitrate solution. Primarily, the change in colour of the reaction mixture was detected by visual observation (Fig. 4). Originally, the AgNO_3_ solution and initial “hairy” root extracts were colourless. The colour of AgNO_3_ turned into a brick-dark color with the addition of the “hairy” root extracts. The colour change started 10–30 min after the AgNPs initiation procedure. Moreover, the rapid browning of the solutions was detected in the variants with the heated aqueous extracts. The solution colour changed gradually from yellow to dark brown in 24 h of contact time, indicating the preliminary formation of AgNPs. The variation in the AgNPs size and shape can affect the change in the colour of the colloidal solution. The colour of the colloidal solution was a persistent bright-brown and stable for 12 months (Fig. S3†), suggesting that both the synthesis of AgNPs was complete and that the mature structures of the nanoparticles were obtained. Thus, according to visual observation, both water and ethanol extracts demonstrated the possibility for the synthesis of AgNPs.

**Fig. 5:**
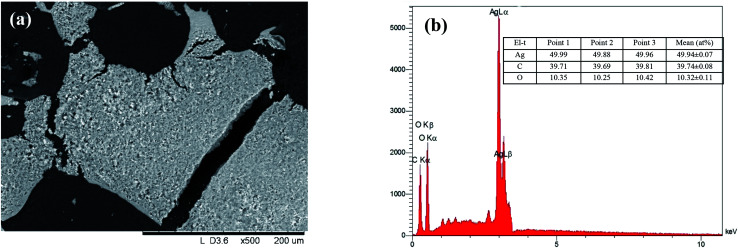
SEM image (a), EDX elemental distribution and analysis (b) of dried AgNPs.

The XRD patterns of the as-prepared AgNPs using “hairy” root extracts clearly show the main peaks at (2*θ*) 38.19, 44.37, 64.56 and 77.47 Bragg reflections, corresponding to the (111), (200), (220) and (311) sets of lattice planes, respectively (Fig. S4†). These peaks manifested the face centered cubic structure of AgNPs (JCPDS 89-3722). The chemical composition of the obtained AgNPs was analyzed by EDX. The EDX spectra of the as-prepared AgNPs revealed a strong signal for silver, carbon and oxygen (Fig. 5). However, there was no signal of nitrogen from AgNO_3_, indicating the formation of bare AgNPs without salt as an impurity. The C and O signals come from the combination of organic compounds of the “hairy” root extract, suggesting that a component of the extract is capping the AgNPs.

**Fig. 6:**
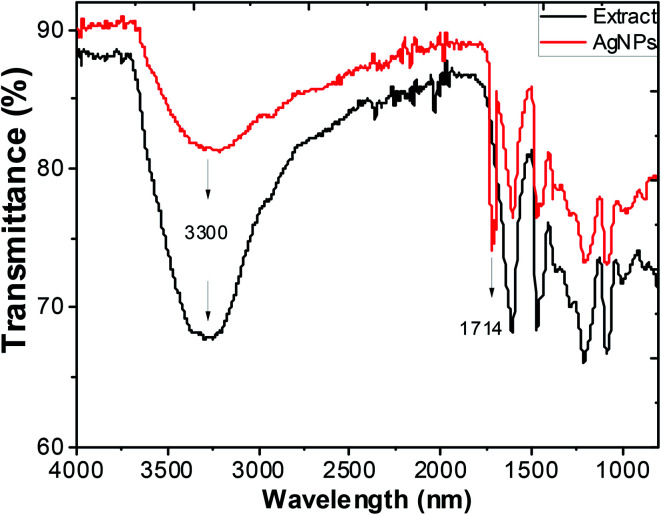
FTIR spectra of ethanol extracts of *Artemisia tilesii* before reaction with Ag^+^ ions, and after reaction and formation of AgNPs.

Typical FTIR absorption spectra in the region of 400–4000 cm^−1^ of the ethanol extract of “hairy” roots before and after bioreduction of Ag^+^ ions are shown in Fig. 6.

Absorbance bands in Fig. 6 were observed at 3300, 2942, 1600, 1456, 1362, 1290, 1216, 1091 and 1031 cm^−1^. The peaks of the stretching vibration of the bound hydroxyl group, intermolecular hydrogen bond and the C=C stretch vibration of the aromatic rings of the extract were represented at 3300 cm^−1^, 1600 cm^−1^ and 1456 cm^−1^, respectively. The absorbance bands at 1330–1031 cm^−1^ are associated with the stretching vibrations for C–O (esters, ethers and polyols), respectively. In particular, the 1216 cm^−1^ band arises most probably from the C–O group of polyols such as flavonoids and terpenes. The peak due to the NO_3_^−^ ions of the initial AgNO_3_ in the final colloidal solution of AgNPs was not observed (usually at 1380 cm^−1^),^53^ and this result was consistent with the EDX measurements. The intensive absorption peak near 1714 cm^−1^ in the AgNPs solution demonstrates the characteristic bands of the C=O bonds. The appearance of this band may be due to the fact that the several components of “hairy” roots extract are mainly oxidized to carbonyl-derivates. Thus, this peak can be used to indicate that the synthesis of AgNPs involves the bioreduction of the AgNO_3_ solution.

**Scheme 1:**
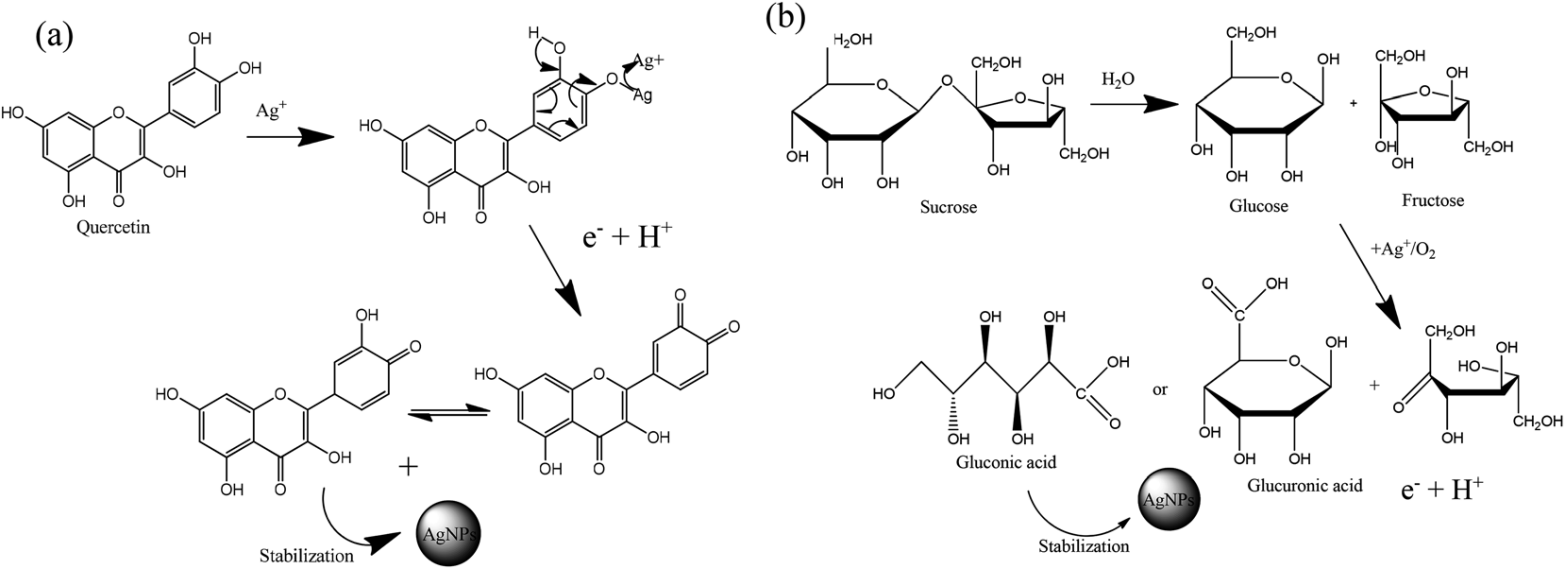
Estimation of the reaction mechanism of the transformation from Ag^+^ to AgNPs by bioreduction (on the example quercetin (a) and sucrose (b)).

Sucrose and polyphenols seem to play the main role in Ag^+^ ion bioreduction.^39^ The possible schematic mechanism of the bioreduction reaction of synthesized AgNPs using the ethanol extract of “hairy” roots is presented in Scheme 1.

The interaction of biomolecules containing hydroxyl groups with high reducing activity can reduce Ag^+^ ions *via* intermediate-complex formation, followed by oxidation through hydrogen abstraction, which initially yields a hydrate (Scheme 1). In these transformations, two stages of AgNPs formation can be recognized:

(1) the nucleation stage, where the Ag^0^ atoms form a small shell (or nuclei);

(2) the growth stage, in which the small nuclei of the metal are grouped, giving rise to the formation of NPs.^20^

On the other hand, Ag^+^ could initiate a sequence of reactions leading to the reactive species formation: 
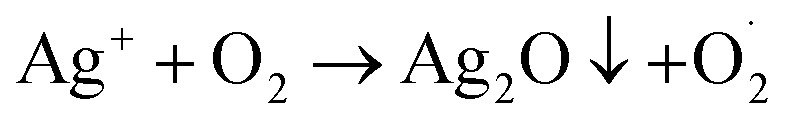
. In addition, the superoxide radical 
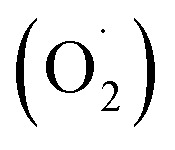
 could then react with bioactive compounds such as flavonoids, and this process results in obtaining products of oxidation or decomposition (Scheme 1). Finally, the natural biomacromolecules obtained in the reaction mixture with linear and branched structures would save the shell AgNPs, creating a protective core from growth as stabilizing agents for the nanoparticles.^20^ This is because the Ag^+^ ion near the AgNPs surface can initiate a complex with molecules containing electron donor groups (O,N-containing motives) that are usually present in flavonoids, phenolic and nucleic acids. Thus, the synthesized AgNPs can be stable without additional chemical reagents.

**Fig. 7:**
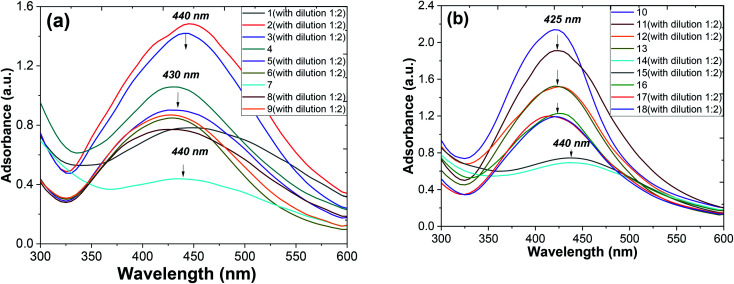
UV-vis spectra of AgNPs obtained from *Artemisia annua* (a) and *A. tilesii* (b) “hairy” root clones with various extraction conditions: EtOH (70 vol%), H_2_O at 28 °C, and water extraction at 28 °C with additional heating at 80 °C.

Metallic nanoparticles (*e.g.*, silver, gold) display characteristic optical absorption spectra in the longwave UV region (315–400 nm) and visible range (400 to 800 nm) called surface plasmon resonance (SPR).^10^ The absorbance peak usually arises as a result of the excitation of the localized SPR oscillations of the conduction electrons in the case of noble nanoparticles, which is not similar to the AgNO_3_ solution (330 nm). The checking AgNPs formation was monitored by the specific absorption band in the UV spectra under the visible range 400–450 nm (Fig. 7). A broadened peak at 440 nm corresponds to the transverse SPR typically observed for nonisotropic AgNPs.^11^

The UV-vis spectra of the colloidal solutions of AgNPs obtained using *A. annua* and *A. tilesii* “hairy” roots were similar, and the nanoparticles were synthesized by *A. annua* and *A. tilesii* extracts. However, a notable difference in the peak intensity was found between the peaks presented in parts (a) and (b) of Fig. 7. The remarkably high intensity of the peak indicates that the size distribution of the particles is narrow.

**Fig. 8:**
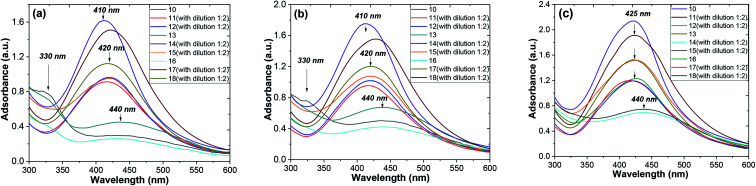
UV-vis absorption spectra of AgNPs obtained by *Artemisia tilesii* “hairy” root clones [no. 17, 21 and 22] with various extraction conditions: 10, 13 and 16 – EtOH (70 vol%), 11, 14 and 17 – aqueous extract at 28 °C, and 12, 15 and 18 – aqueous extract at 28 °C and additional heating at 80 °C: 1 day (a), 2 days (b) and 10 days (c).

At the same time, from the shape of the absorption peak, we can also acquire information on the changes of the AgNPs size distribution. As is well known,^54^ when the solution system is monodisperse (narrow size distribution), the peak shape is symmetric and the value of the full width at half-maximum (FWHM) is small. When the system is polydisperse, the peak shape is asymmetric, which suggests that the peak actually consists of two or more absorption peaks. Thus, the broadening of the peak at 420–440 nm (Fig. 8) indicates that the particles are polydispersed.^55^ The peak shape changes visibly from wide asymmetric for the ethanol extracts to symmetric for the aqueous ones. In addition, the absorption peak became narrower with a decrease in the corresponding FWHM values. The absorbance was greater in the aqueous (80 °C) extracts compared to the ethanol one. Those results mean that the size distribution becomes narrower, and the colloid system changes from polydispersion to monodispersion. This effect can also be seen in the following TEM results.

Fig. 6 shows the shifts of the peak positions and the shapes of the absorption spectra during the whole reaction process at time intervals of 1, 2 and 10 days for the ethanol and aqueous extracts. When the injection of the AgNO_3_ solution was finished, a very broad UV-visible band (see Fig. 8a) appeared at 410–440 nm. It is not similar to AgNO_3_ (330 nm) or the extract solution absorption spectrum, showing that the particles were really formed at that time. When the reaction continued for a duration of 2 days, a remarkable peak appeared and the peak position changed from 440 nm on the first day to 420 nm on the second day, as seen in Fig. 8b and c. So, in the AgNPs formation process, large particles were formed at the beginning of the reaction. Then, the large particles were decomposed to small ones. Later on, the position changes of the corresponding peaks kept the blue shift, but rather did not turn to the red shift from 425 nm to 410 nm at 10 days (see Fig. 8c). It was observed that the peak was blue shifted in the absorption spectrum from 425 to 420 nm with increasing reaction time (1, 2 and 10 days).^56^ The intensity of the absorption bands increased as the reaction time progressed. After the optimized reaction time (2 days) with considerable intensity of the bands, the maximum formation of AgNPs was achieved.

Thus, the growth process of the AgNPs capped by “hairy” root extracts can be summarized into the following stages: (1) large nanoparticles were formed in a rapid nucleation process during the first 15 min; (2) the large nanoparticles were rapidly decomposed into small particles in the following 2 days; (3) a longer time (10 days) was required for a slight increase of the core size after the big particles decomposed into smaller ones. In addition, during the whole formation process of the nanoparticles, the system changed from a phase that was polydisperse in size to one that was monodisperse in size. Thus, during the formation process of the nanoparticles, the particle size distribution becomes narrow.

**Fig. 9:**
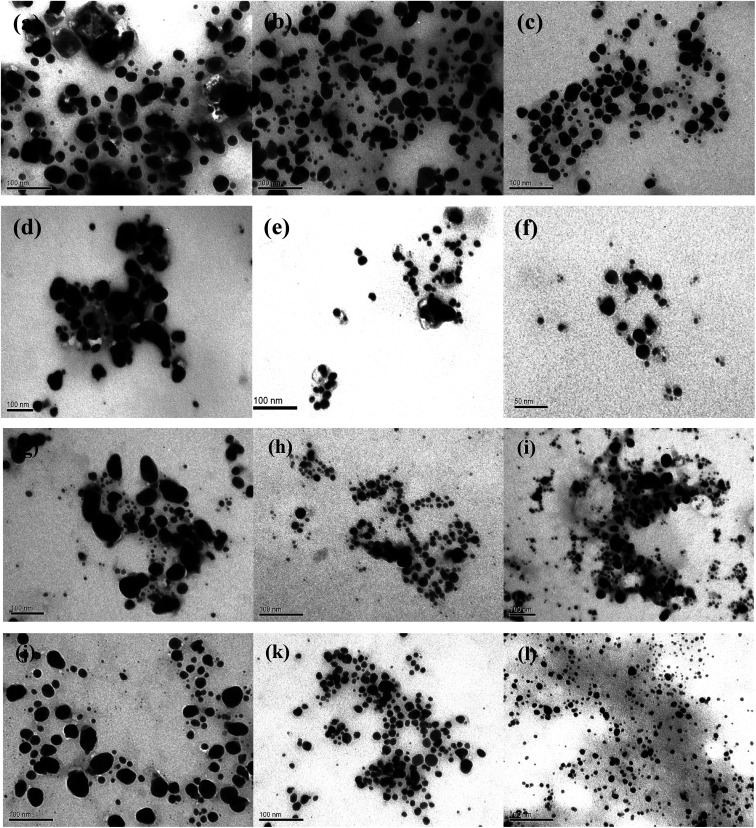
TEM images of AgNPs synthesis from *Artemisia annua* (a–f) and *A. tilesii* (g–l) “hairy” root clones: [no. 15] control (a–c) and [no. 19] transgene (d–f); [no. 22] (g–i) and [no. 21] (j–l) with EtOH (70 vol%) (a, d, g and j), aqueous solution obtained at 28 °C (b, e, h and k), and using additional heating at 80 °C (c, f, i and l).

The morphology of AgNPs was observed by TEM microscopy of the samples obtained by using different *A. annua* and *A. tilesii* “hairy” root extracts (Fig. 9).

Nanoparticles obtained using several root extracts differed by their form (spherical, oval) and size (from 5 to 100 nm) (Fig. 9). In the ethanol extract, the primary irregular and assembly AgNPs with huge nanoparticle size distributions (5–50 nm) and random shapes were formed by reducing Ag^+^ by the compounds extracted from the roots (Fig. 9a, d, g and j). In the case of using the first type of aqueous solution (obtained at 28 °C), such irregular nanoparticles formed multipod-like anisotropic (spherical, triangles) structures that were 10–20 nm in size (Fig. 9b, e, h and k). When the second type of aqueous extract was used (the extracts additionally heated at 80 °C), smaller aggregate nanoparticles with spherical, oval, and triangular forms were obtained (Fig. 9c, f, i and l). Thus, after using the first type of aqueous extract, the particle size strongly decreased to 5–20 nm compared to the NPs size in the case of using the ethanol extracts. In the case of using the second type of aqueous extracts, the size of the nanoparticles slightly decreased to 5–10 nm. In this variant, the particles were more uniform than those obtained by the firs type of aqueous extracts. The smallest (5.0 ± 0.5 nm) and regular particles were obtained by *A. tilesii* second type aqueous extract (Fig. 9). The TEM micrograph demonstrate that NPs initiated after the addition of *A. annua* “hairy” root extracts make up ones with high diameter range, especially in ethanol extracts.

The results of the TEM and UV analyses are consistent with each other. The TEM data demonstrate that the *A. annua*-initiated nanoparticles were quite big, and were in the range of 30–40 nm for the ethanol extracts with broad size distribution. The *A. annua* and *A. tilesii* root ethanol extracts were characterized by different reducing powers (Fig. 3). The greater activity was manifested for the *A. tilesii* extracts compared to the *A. annua* extracts. This unique activity may cause differences in the particle sizes and other characteristics of AgNPs synthesized after the extracts were added to the AgNO_3_ solution. For example, using the extracts with the high reducing power (*e.g.*, *A. tilesii* second type aqueous extracts) resulted in the formation of the smallest nanoparticles. However, the size of the particle is a critical parameter for the inhibition of cell growth in the process of antimicrobial application of AgNPs. The smaller nanoparticles have more antibacterial and antimicrobial activity due to providing more surface exposure to the bacterial membrane. This can be the key to understanding why the other investigations were studied with *A. tilesii* “hairy” root extracts.

### 3.3 Antimicrobial activity of the AgNPs

The antimicrobial activity of the synthesized AgNPs was evaluated by using the standard disk diffusion method.^37^ The microorganisms were cultivated in Petri dishes with solidified nutrient agar medium (Fig. S5†). AgNPs were added in concentrations of 20 μl per disk, and ciprofloxacin was used as a positive control. The obtained inhibition zones indicate greater antimicrobial activity of the AgNPs sample compared to the AgNO_3_ solution. The antimicrobial activity of the tested compounds with the zone of inhibition are represented in Table 2.

**Table 2:** Antimicrobial activity of the tested samples against test microorganisms^a^

Tested compounds	Zone of inhibition (mm)			
	*C. albicans*	*S. aureus*	*P. aeruginosa*	*E. coli*
Ciprofloxacin	0	21.3 ± 1.3	28.7 ± 1.3	29 ± 1.1
AgNPs (no. 16)	10.0 ± 0.1	12.3 ± 0.6	10.3 ± 0.6	12.0 ± 0.1
AgNPs (no. 17)	11.0 ± 1.1	14.3 ± 0.6	8.3 ± 0.6	13.1 ± 0.1
AgNPs (no. 18)	14.6 ± 0.6	16.7 ± 0.7	13.3 ± 0.6	12.6 ± 0.6
AgNO_3_ (solution)	11.0 ± 0.1	11.6 ± 0.7	0	10.3 ± 0.6

a Values are means of triplicate determination ± standard deviations.

Hence, the results revealed that the AgNPs efficiently suppressed the microorganisms (Table 2). The AgNPs (no. 18) with low size (1–20 nm) showed the maximal antimicrobial activity for all tested microorganisms. The decrease in the AgNPs size can lead to an increase in their ability for cell membrane effect due to the higher surface activity, and thus improving antimicrobial activity. It is possible that AgNPs stabilized by extract components (such as sucrose or polyols) interacted with the building blocks of the surfaces of bacteria (*e.g.*, disaccharide-pentapeptide composed (amino sugars^57^)) and disrupted the functions of the bacterial cells (*e.g.*, respiration), which led to microbial cell death. Similar types of results have also been reported earlier.^58^ In addition, the inhibition effect of all obtained AgNPs was higher for *S. aureus* than for *E. coli*. Conversely, for most of the AgNPs described in the literature, the opposite result is observed.^59^ The cell wall of *S. aureus* (Gram-positive bacterium) contains a thick and rigid peptidoglycan layer, and *E. coli* (Gram-negative bacterium) has a thin peptidoglycan layer in the cell wall.^59^ This structure can affect the sensitivity of the bacterial cells to different antimicrobial agents. At the same time, the mechanism of the antimicrobial activity of AgNPs is complicated and not fully studied now. It should be noted that, in contrast to antibiotic (effective for Gram-negative and Gram-positive bacteria), the studied substances were effective not only against bacteria, but also *C. albicans*. Thus, the obtained AgNPs were also characterized by antifungal properties.

So, the results of our study confirm that the “hairy” root cultures of *Artemisia* spp. medicinal plants can be used for the effective “green” synthesis of AgNPs, which are characterized by their small size and antimicrobial activity.

## 4 Conclusions

In summary, we reported a one-pot procedure for the effective extraction of bioactive compounds and reproducible “green” synthesis of AgNPs using “hairy” root extracts. The novelty of this work is in the fact that bioactive silver NPs of small size (5–100 nm) were obtained by a “green” synthesis process using extracts from the “hairy” roots of *Artemisia* spp. characterized by the high reducing activity. The NPs with different shapes and sizes can be synthesized by spontaneous self-reduction in the presence of bioactive components of *Artemisia annua* and *A. tilesii* “hairy” root extracts. A blue shift of the maximum absorption peak position of the UV-vis spectra occurred at the beginning of the reaction, followed by a red shift. In the AgNPs formation process, large particles were formed at the beginning of the reaction, and then they decomposed to small ones. The morphology of the AgNPs was found to be spherical or irregular. The UV-visible spectra of the final AgNPs colloidal phase show that the system was monodisperse, and can remain stable for a long time (12 months). In the work, with the aid of the UPLC-ESI-UHR-Qq-TOF-MS analysis, 17 compounds were identified in the root extracts, including polyphenols and terpenoids. Most of the compounds were identified as having potential for antioxidant, anti-inflammatory, hepatoprotective, cardioprotective, antiamyloidogenic and anticancer therapy, and cosmetics. In the present study, the antimicrobial activity of the “green” synthesized AgNPs were assayed against Gram-positive, Gram-negative bacteria and *C. albicans*. The results indicate the higher activity of AgNPs compared to the aqueous AgNO_3_ solution was found in the test with *P. aeruginosa*. The data testify the possibility of using *Artemisia* spp. “hairy” root extracts (characterized by the increased reducing activity) for the fast and effective synthesis of a stable AgNPs solution with antimicrobial activity.

## Conflicts of interest

The authors declare that they do not have competing interests.

## Supplementary Material

RA-010-D0RA07784D-s001

## References

[cit1] Lloyd J. R., Byrne J. M., Coker V. S. (2011). Curr. Opin. Biotechnol..

[cit2] Patra S., Mukherjee S., Kumar A., Ganguly A., Sreedhar B., Ranjan C. (2015). Mater. Sci. Eng., C.

[cit3] Dung Dang T., Thu Tuyet Le T., Fribourg-Blanc E., Chien Dang M. (2011). Adv. Nat. Sci.: Nanosci. Nanotechnol..

[cit4] Sethuraman G. , Shim J. and Lee Y. R., Microwave assisted green synthesis of fluorescent N-doped carbon dots: cytotoxicity and bio-imaging applications, Elsevier, 2016 10.1016/j.jphotobiol.2016.05.017 27236237

[cit5] Fouda M. M. G., Abdelsalam N. R., El-naggar M. E., Zaitoun A. F., Salim B. M. A., Bin-jumah M., Allam A. A., Abo-marzoka S. A., Kandil E. E. (2020). Int. J. Biol. Macromol..

[cit6] Ghoreishi S. M., Behpour M., Khayatkashani M. (2011). Phys. E.

[cit7] Hoek E. M. V., Marambio-Jones C. (2010). J. Nanopart. Res..

[cit8] Wang H., Qiao X., Chen J., Ding S. (2005). Colloids Surf., A.

[cit9] Khatoon U. T., Rao G. V. S. N., Mantravadi K. M., Oztekin Y. (2018). RSC Adv..

[cit10] Zaarour M., El Roz M., Dong B., Retoux R., Aad R., Cardin J., Dufour C., Gourbilleau F., Gilson J. P., Mintova S. (2014). Langmuir.

[cit11] Patil M. P., Do Kim G. (2017). Appl. Microbiol. Biotechnol..

[cit12] Begum N. A., Mondal S., Basu S., Laskar R. A., Mandal D. (2009). Colloids Surf., B.

[cit13] Kharissova O. V., Dias H. V. R., Kharisov B. I., Pérez B. O., Pérez V. M. J. (2013). Trends Biotechnol..

[cit14] Nam G., Rangasamy S., Purushothaman B., Song J. M. (2015). Nanomater. Nanotechnol..

[cit15] Kuppusamy P., Ichwan S. J. A., Parine N. R., Yusoff M. M., Maniam G. P., Govindan N. (2015). J. Environ. Sci..

[cit16] Sinha S. N., Paul D., Halder N., Sengupta D., Patra S. K. (2015). Appl. Nanosci..

[cit17] Ahmed S., Ahmad M., Swami B. L., Ikram S. (2016). J. Adv. Res..

[cit18] Rafique M., Sadaf I., Rafique M. S., Tahir M. B. (2017). Artif. Cells, Nanomed., Biotechnol..

[cit19] Saini P., Saha S. K., Roy P., Chowdhury P., Sinha Babu S. P. (2015). Exp. Parasitol..

[cit20] Park J. W., Shumaker-Parry J. S. (2014). J. Am. Chem. Soc..

[cit21] Park J. (2015). ACS Nano.

[cit22] Dhand V., Soumya L., Bharadwaj S., Chakra S., Bhatt D., Sreedhar B. (2016). Mater. Sci. Eng., C.

[cit23] Chandra S. (2012). Biotechnol. Lett..

[cit24] El-Esawi M. A., Elkelish A., Elansary H. O., Ali H. M., Elshikh M., Witczak J., Ahmad M. (2017). Oxid. Med. Cell. Longevity.

[cit25] Dilshad E., Cusido R. M., Estrada K. R., Bonfill M., Mirza B. (2015). PLoS One.

[cit26] Bulgakov V. P. (2008). Biotechnol. Adv..

[cit27] Sevón N., Oksman-Caldentey K. M. (2002). Planta Med..

[cit28] Giri A., Narasu M. L. (2000). Biotechnol. Adv..

[cit29] Hussain M. S., Fareed S., Ansari S., Rahman M. A., Ahmad I. Z., Saeed M. (2012). J. Pharm. BioAllied Sci..

[cit30] Matvieieva N. A., Morgun B. V., Lakhneko O. R., Duplij V. P., Shakhovsky A. M., Ratushnyak Y. I., Sidorenko M., Mickevicius S., Yevtushenko D. P. (2020). Plant Physiol. Biochem..

[cit31] Griffin D. (2001). J. Ethnobiol..

[cit32] Drobot K. O., Matvieieva N. A., Ostapchuk A. M., Kharkhota M. A., Duplij V. P. (2017). Prep. Biochem. Biotechnol..

[cit33] Matvieieva N., Drobot K., Duplij V., Ratushniak Y., Shakhovsky A., Kyrpa-Nesmiian T., Mickevičius S., Brindza J. (2019). Prep. Biochem. Biotechnol..

[cit34] Al-Shalabi Z., Doran P. M. (2016). J. Biotechnol..

[cit35] Pękal A., Pyrzynska K. (2014). Food Anal. Methods.

[cit36] Chan S. G., Murphy P. A., Ho S. C., Kreiger N., Darlington G., So E. K. F., Chong P. Y. Y. (2009). J. Agric. Food Chem..

[cit37] Hudzicki J. (2016). Am. Soc. Microbiol..

[cit38] Stalikas C. D. (2007). J. Sep. Sci..

[cit39] Pietta P. G. (2000). J. Nat. Prod..

[cit40] Laghari A. H., Memon S., Nelofar A., Khan K. M., Yasmin A. (2011). Food Chem..

[cit41] Wani H., Shah S., Banday J. (2014). J. Phytopharm..

[cit42] Tu Y. , From *Artemisia annua* L. to Artemisinins. The Discovery and Development of Artemisinins and Antimalarial Agents, Academic Press Agents, Elsevier, 2017

[cit43] Shi P., Fu X., Liu M., Shen Q., Jiang W., Li L., Sun X., Tang K. (2017). Plant Cell, Tissue Organ Cult..

[cit44] Mahfoudhi A., Prencipe F. P., Mighri Z., Pellati F. (2014). J. Pharm. Biomed. Anal..

[cit45] Stintzing F. C., Kammerer D., Schieber A., Adama H., Nacoulma O. G., Carle R. (2004). Z. Naturforsch., C: J. Biosci..

[cit46] Aurang Zeb M. (2017). Pharm. Pharmacol. Int. J..

[cit47] Salehi B., Venditti A., Sharifi-Rad M., Kręgiel D., Sharifi-Rad J., Durazzo A., Lucarini M., Santini A., Souto E. B., Novellino E., Antolak H., Azzini E., Setzer W. N., Martins N. (2019). Int. J. Mol. Sci..

[cit48] Kasiri N., Rahmati M., Ahmadi L., Eskandari N. (2018). Inflammopharmacology.

[cit49] Galasso S., Pacifico S., Kretschmer N., Pan S. P., Marciano S., Piccolella S., Monaco P., Bauer R. (2014). Phytochemistry.

[cit50] Wang J., Ren H., Xu Q. L., Zhou Z. Y., Wu P., Wei X. Y., Cao Y., Chen X. X., Tan J. W. (2015). Food Chem..

[cit51] Kim J. K., Park S. U. (2018). EXCLI J..

[cit52] Naveed M., Hejazi V., Abbas M., Kamboh A. A., Khan G. J., Shumzaid M., Ahmad F., Babazadeh D., FangFang X., Modarresi-Ghazani F., WenHua L., XiaoHui Z. (2018). Biomed. Pharmacother..

[cit53] Silverstein R. M. , Webster F. X. and Kiemle D. J., Spectrometric Identification of Organic Compounds, John Wiley & Sons, 7th edn, 2005

[cit54] Brown K. R., Walter D. G., Natan M. J. (2000). Chem. Mater..

[cit55] Sheny D. S., Mathew J., Philip D. (2011). Spectrochim. Acta, Part A.

[cit56] Wiley B., Herricks T., Sun Y., Xia Y. (2004). Nano Lett..

[cit57] Scheffers D., Pinho M. G. (2005). Microbiol. Mol. Biol. Rev..

[cit58] Kvitek L., Vanickova M., Panacek A., Soukupov J., Dittrich M., Valentova E., Prucek R., Bancirova M., Milde D., Zboril R. (2009). J. Phys. Chem. C.

[cit59] Ahmad T., Wani I. A., Manzoor N., Ahmed J., Asiri A. M. (2013). Colloids Surf., B.

