# Supplementary material for: ‘Hairy’ root extracts as source for ‘green’ synthesis of silver nanoparticles and medical applications
Source: RSC Adv. 2020 Oct 27;10(65):39434–46. doi: 10.1039/d0ra07784d (PMC9057394; doi:10.1039/d0ra07784d)
Supplement: RA-010-D0RA07784D-s001 [file RA-010-D0RA07784D-s001.pdf]

Electronic Supplementary Material (ESI) for RSC Advances.
This journal is © The Royal Society of Chemistry 2020

Supplementary materials

'Hairy' root Extracts as Source for 'Green' Synthesis of Silver Nanoparticles and Medical Applications

Natalia Kobylinska[1], Anatolij Shakhovsky[2], Olena Khainakova[3], Dmytro Klymchuk[4], Liliya Avdeeva[5], Yakiv Ratushnyak[2], Volodymyr Duplij[2], Nadiia Matvieieva[2]

[1]*Taras Shevchenko National University of Kyiv, 64 Volodymyrska Str., Kyiv, 01601, Ukraine*
[2]*Institute of Cell Biology and Genetic Engineering, NAS of Ukraine, 148 Zabolotnogo Str, Kyiv, 03143, Ukraine*
[3]*University of Oviedo, 8 Julián Claveria Av., Oviedo, 33006, Spain*
[4]*M.G.Kholodny Institute of Botany, NAS of Ukraine, 2 Tereshchenkivska* Str, *Kyiv, 02000, Ukraine*
[5]*Zabolotny Institute of Microbiology and Virology, NAS of Ukraine, 154 Zabolotnogo Str, Kyiv, 03143, Ukraine*

Corresponding e-mail: kobilinskaya@univ.kiev.ua

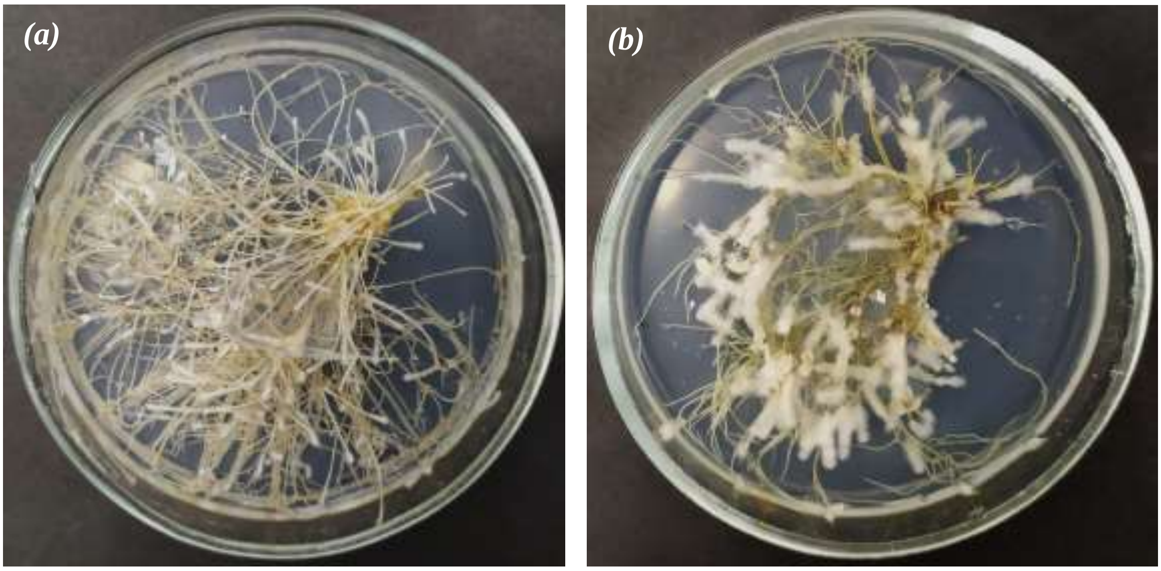

Fig. S1. Images of *Artemisia annua* (a) and *Artemisia tilesii (b)* “hairy” root clones

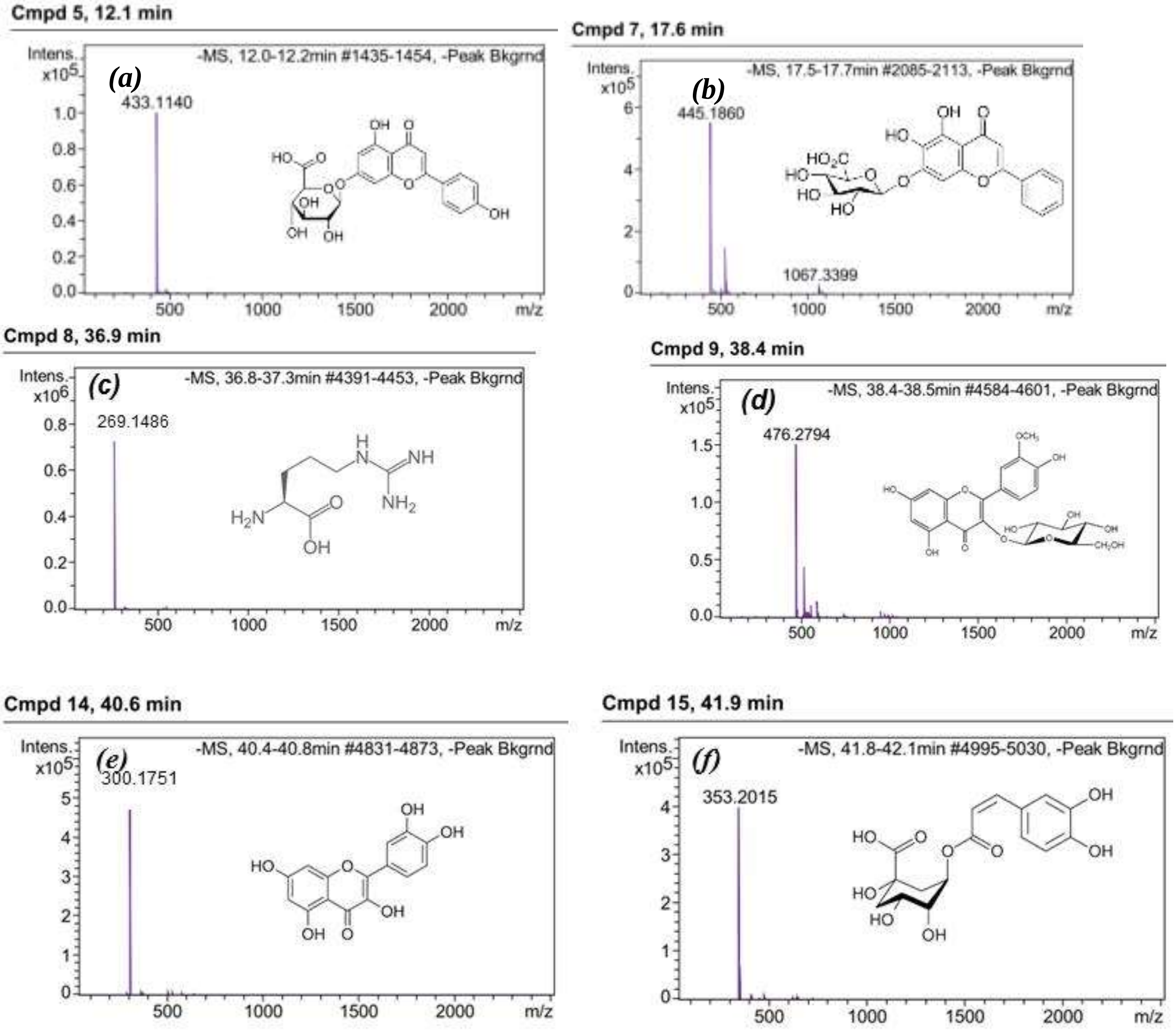

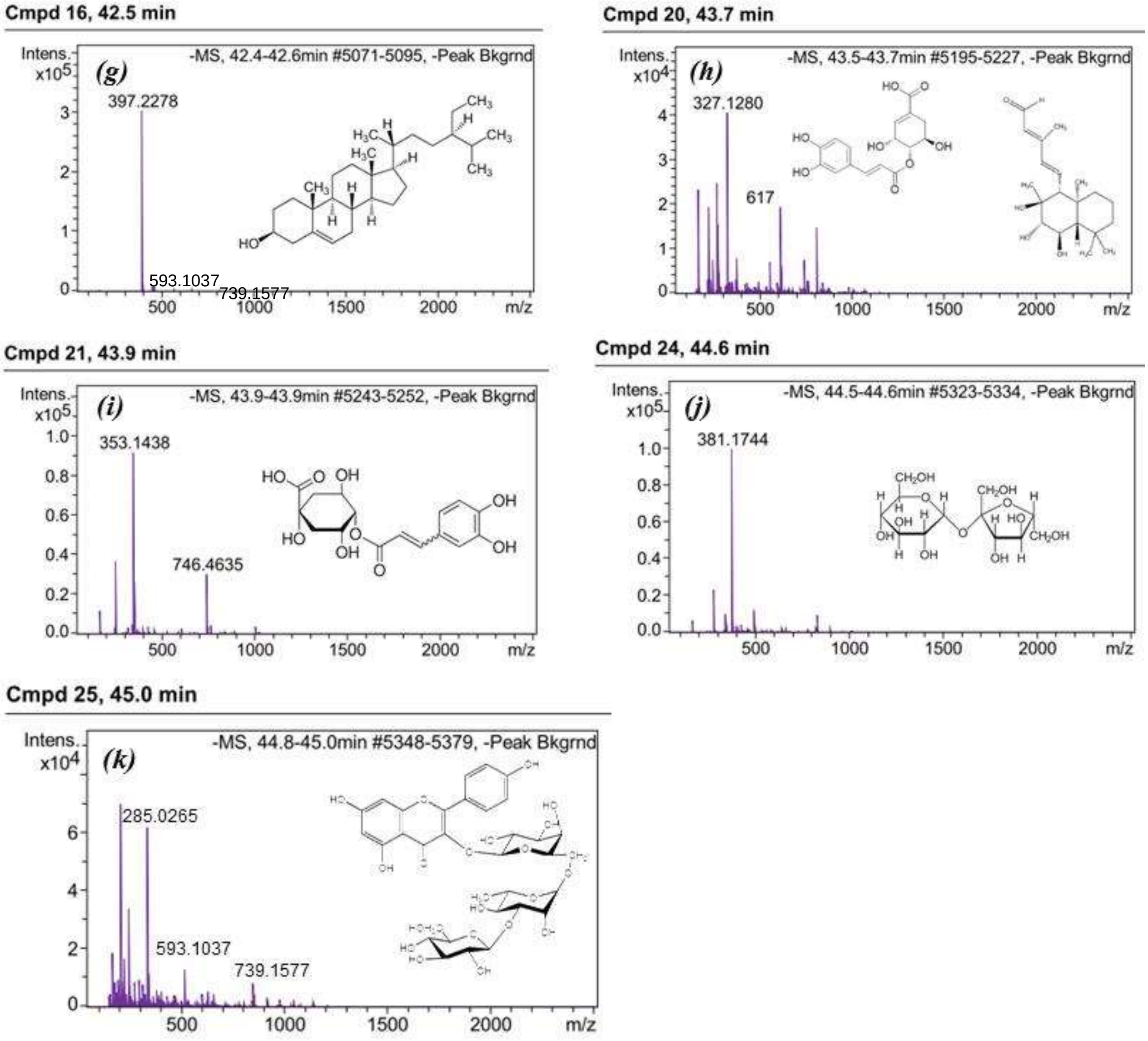

Fig. S2. Fragmentation behavior of the ethanol extracts of *A. tilesii* roots (*a* - Apigenin-7-O-glucoside; *b* - Baicalein-7-O-glucuronide; *c* – Arginin; *d* - Isorhamnetin 3-O-glucoside; e – Quercetin; f - Chlorogenic acid; *g* – Sitosterol; *h* - Caffeoylshikimic acids and Sterebin J/Sterebin I; *i* - Caffeoylquinic acid; *j* - Sucrose+K^+; *k* - kaempferol-3-O-galactoside-rhamnoside-7-O-rhamnoside) MS in negative ionization mode.

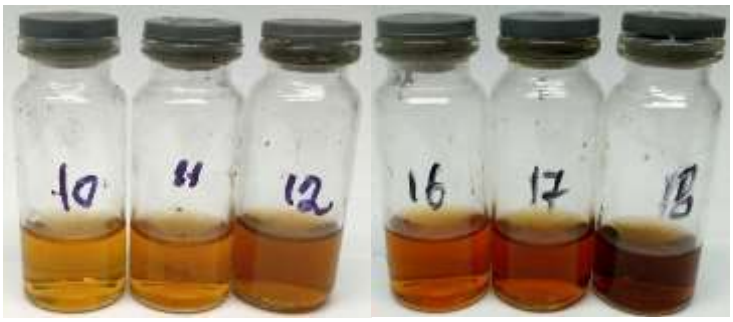

Fig. S3. Stability of AgNPs in six month after the initiation by *Artemisia tilesii* “hairy” root extracts

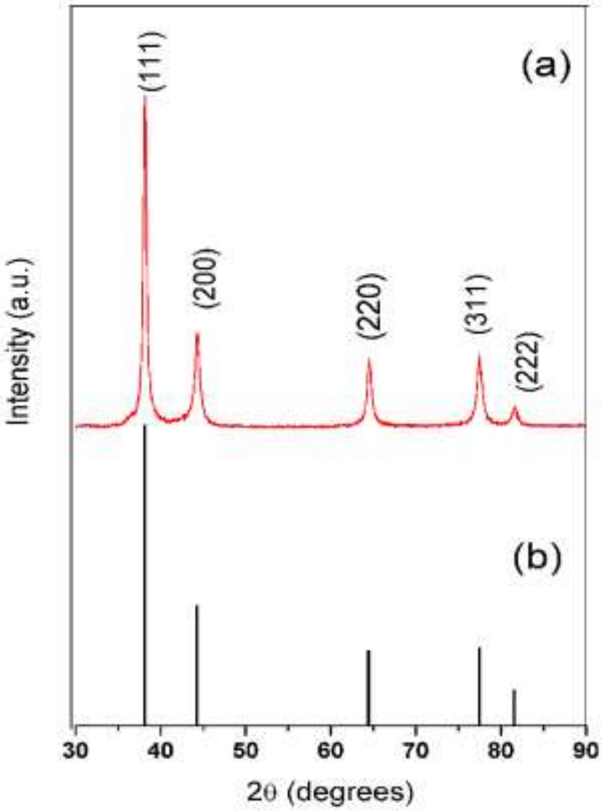

Fig. S4. XRD pattern of prepared AgNPs by *Artemisia tilesii* "hairy" root extracts (a) and JCPDS file No. 89-3722 (b)

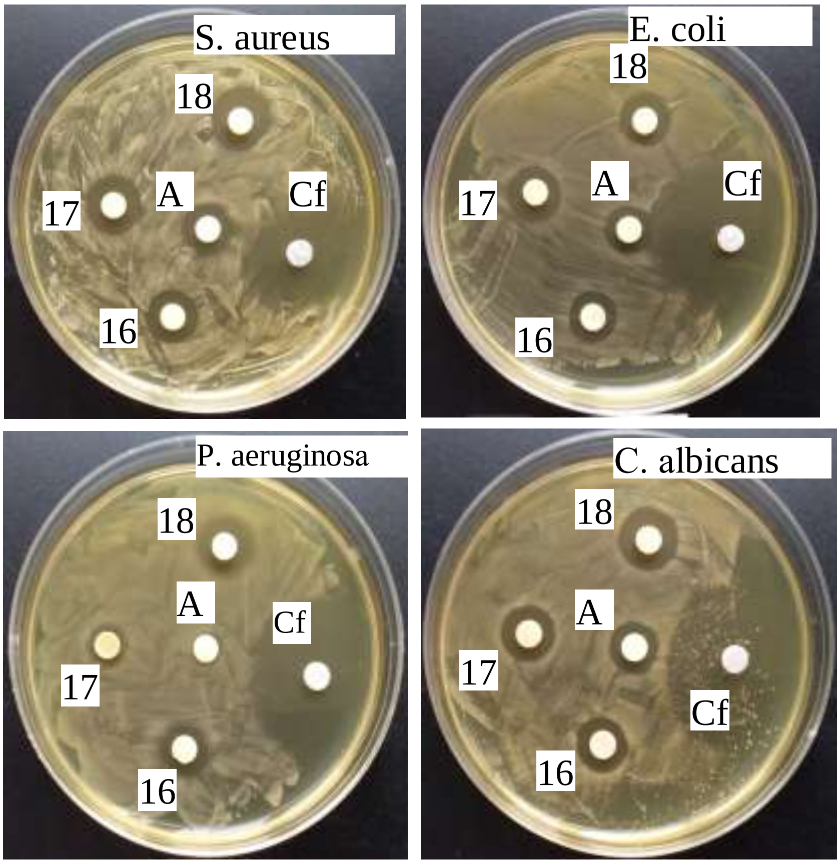

Fig. S5. Antimicrobial effect of AgNPs obtained from A. *tilesii* "hairy" root clones [*22k*: 16, 17,18], $AgNO_3$(solution) and antibiotic on *St. aureus, Candida albicans, Pseudomonas Aeruginosa and Escherichia coli.*
